# Ketamine Analog Methoxetamine Induced Inflammation and Dysfunction of Bladder in Rats

**DOI:** 10.3390/ijms18010117

**Published:** 2017-01-18

**Authors:** Qiang Wang, Qinghui Wu, Junpeng Wang, Yang Chen, Guihao Zhang, Jiawei Chen, Jie Zhao, Peng Wu

**Affiliations:** 1 Department of Urology, Nanfang Hospital, Southern Medical University, Guangzhou 510515, China; doctorwangqiang@gmail.com (Q.W.); doctor_wqh@126.com (Q.W.); wangjunpeng1990@126.com (J.W.); cyhm28@sina.com (Y.C.); jyzhanggh@163.com (G.Z.); miwaicjw@163.com (J.C.); 2 School of Pharmaceutical Sciences, Southern Medical University, Guangzhou 510515, China

**Keywords:** methoxetamine, bladder dysfunction, cystitis, cytokines, ketamine

## Abstract

The novel synthetic psychoactive ketamine analog methoxetamine is reportedly being used for recreational purposes. As ketamine use can result in urinary dysfunction, we conducted the present study to investigate how methoxetamine affects the bladder. A cystometry investigation showed that female Sprague-Dawley rats experienced increased micturition frequency bladder dysfunction after receiving a daily intraperitoneal injection of 30 mg/kg methoxetamine or ketamine for periods of 4 or 12 weeks. Histologic examinations of rat bladder tissue revealed damaged urothelium barriers, as well as evidence of inflammatory cell infiltration and matrix deposition. The drug-treated rats showed significantly upregulated levels of pro-inflammatory cytokines such as IL-1β, IL-6, CCL-2, CXCL-1, CXCL-10, NGF, and COX-2. In addition, interstitial fibrosis was confirmed by increased levels of collagen I, collagen III, fibronectin and TGF-β. Besides direct toxic effect on human urothelial cells, methoxetaminealso induced the upregulation related cytokines. Our results indicate that long term methoxetamine treatment can induce bladder dysfunction and inflammation in rats. Methoxetamine was confirmed to produce direct toxic and pro-inflammatory effects on human urothelial cells. Methoxetamine-associated bladder impairment may be similar to ketamine-induced cystitis.

## 1. Introduction

Methoxetamine (MXE) is a structural analog of ketamine (KET), which serves as a *N*-methyl-d-aspartate receptor antagonist [1]. MXE binds to serotonin transporter to suppress the re-uptake of 5-hydroxytryptamine and ensure proper levels of 5-hydroxytryptamine in the brain. MXE has also been shown to increase dopamine secretion and suppress dopamine re-uptake [2]. MXE has only recently been studied as a novel synthetic psychoactive drug, and in 2010, was reported to produce a variety of effects in humans, including feelings of euphoria, calmness, intensive sensations, and physical dissociation, as well as hallucinations and near-death experiences. In 2011, MXE attracted attention for its reported recreational use [3]. Currently, only a limited amount of MXE-related pharmacologic and toxicology data are available and additional studies of its properties and effects are needed. When considering the similarities between MXE and KET, it may be assumed that current MXE abusers include a large number of former KET abusers. In 2007, ketamine was shown to induce a type of severe cystitis [4] that showed a poor response to traditional treatments. The lower urinary tract symptoms (LUTS) continued even when a patient had discontinued ketamine. Furthermore, when upper urinary tract involvement occurs, surgical intervention such as augmentation cystoplasty is required if conservative treatment fails [5]. A series of studies using bladder cell lines, human tissue samples, and animal models have been conducted to examine the biological mechanism of ketamine-induced cystitis. The postulated mechanisms included bladder-epithelial barrier impairment [6], abnormal neurotransmission [7], mast cell activation [8], cell apoptosis [9,10], and oxidative stress [11,12]. Although results of a survey suggested that MXE use may contribute to LUTS development, all of the MXE abusers in that survey had previously used KET at least once [13], and thus the impact of MXE use by itself on LUTS occurrence could not be analyzed. Although Dargan et al. reported histology results showing that MXE produced toxic effects in the bladders and kidneys of mice, further research on how MXE affects the urinary system is necessary [14].

MXE was originally designed and synthesized to function as an antidepressant [5], and was advertised as being less toxic than ketamine to the urinary system [15]. However, additional data are required to verify that MXE is as “bladder friendly” as described. To our knowledge, the effect of MXE on bladder function and inflammation has not been explored in animals. Therefore, we used a rat model of ketamine-induced cystitis to investigate the urodynamic function and histological changes which occur in the bladder after long term MXE and KET treatment.

## 2. Results

### 2.1. Reduced Body Weight and Increased Micturition Frequency Due to Drug Treatment

**Figure 1:**
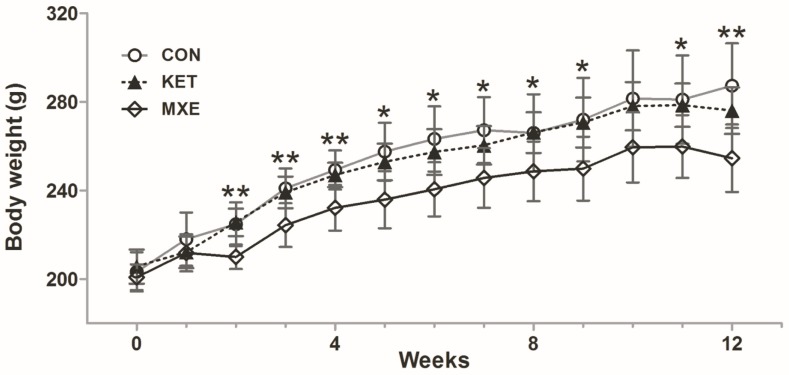
Effect of ketamine and methoxetamine on body weight of rats over a 12-weeks period. Rats were injected with normal saline (CON, *n* = 6), 30 mg/kg methoxetamine (MXE, *n* = 6) or 30 mg/kg ketamine (KET, *n* = 6) for a period of 12 weeks. There were significant differences between the three groups from Week 2 to Week 12 (except at 10 weeks, *p* > 0.05). Data are expressed as the mean ± SD, and compared with one-way ANOVA. * *p* < 0.05, ** *p* < 0.01.

After two weeks of treatment, significant differences in body weight were observed among the three groups of rats: 241.0 ± 9.0 g (control group, as CON group) vs. 224.3 ± 9.9 g (MXE group) vs. 239.1 ± 7.1 g (KET group), *p* = 0.009 (Figure 1). The mean body weight of rats in the MXE group was significantly less when compared with that of rats in the CON group (*p* = 0.008) or KET group (*p* = 0.006). From Week 2 to Week 12, the changes in body weight in the three groups showed similar trends and were statistically significant (except at 10 weeks, *p* = 0.062), suggesting that exposure to methoxetamine could significantly delay weight gain (*p* < 0.05 vs. both the CON group and KET group). In contrast, ketamine showed a negligible effect on body weight (*p* > 0.05).

**Figure 2:**
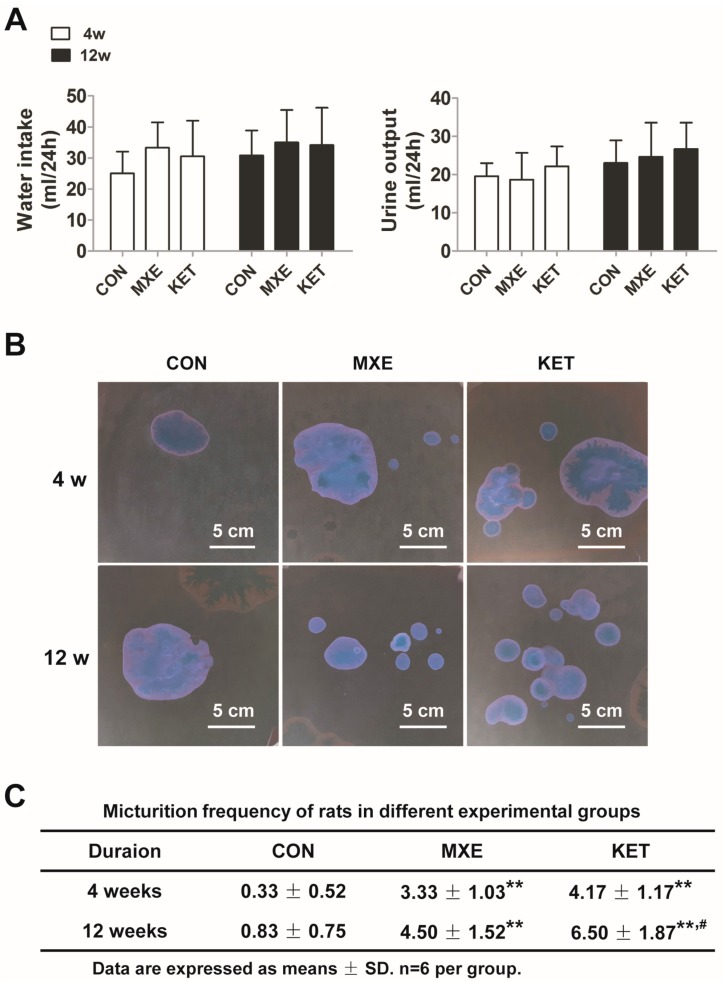
Water intake, urine output and micturition frequencies of rats after 4- and 12-weeks drug treatment. Rats were injected with normal saline (CON, *n* = 6), 30 mg/kg methoxetamine (MXE, *n* = 6) or 30 mg/kg ketamine (KET, *n* = 6) for a period of 12 weeks. (**A**) Changes in the 24h liquid balance in each group were examined after 12 weeks of treatment; (**B**) representative depictions of voiding spot assay showed urination frequency as assessed in the reversed color contrast phase, and analyzed with two-way ANOVA; (**C**) quantification of voiding spot assay (number of voidings per measured 3 h). ** *p* < 0.01 vs. the CON group. ^#^ *p* < 0.05 vs. the MXE group.

As shown in Figure 2A, treatment with either drug did not change the balance between water intake and urine output in rats. Representative graphs of voiding spots in reversed color contrast phase for each group of rats are shown in Figure 2B. An analysis of urination frequency revealed a significant increase in micturition frequency in both the MXE and KET group when compared with micturition frequency in the CON group after 4or 12weeks of treatment (Figure 2C).

### 2.2. Effects of Drug Treatment on Urodynamic Parameters

**Figure 3:**
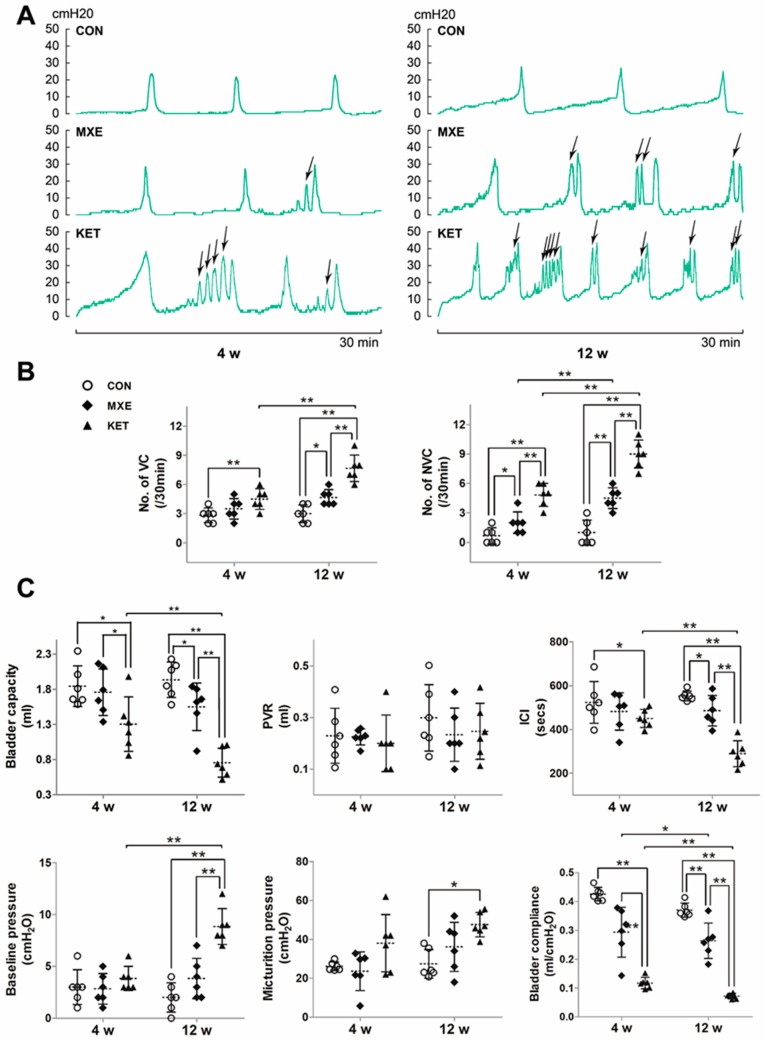
Abnormal voiding patterns of rats due to drug treatment. (**A**) Typical cystometric recordings in the three groups of rats after four or 12 weeks of treatment (30 min, intravesical pressure unit: cm H_2_O, *n* = 6 in each group). The frequency of non-voiding contractions (NVCs) was marked with arrows; (**B**) the frequency of non-voiding contractions (NVCs) was increased in the KET and MXE groups; (**C**) the urodynamic parameters evaluated included voiding contractions (VCs) and non-voiding contractions (NVCs); and bladder capacity, post-void residual (PVR), inter-contraction interval (ICI), baseline pressure, micturition pressure and bladder compliance. Data were analyzed with two-way ANOVA. * *p* < 0.05, ** *p* < 0.01.

Urodynamic examinations were performed to gain further insight into the development of bladder dysfunction due to MXE and KET administration, and representative continuous cystometrograms are shown in Figure 3A. The frequency of non-voiding contractions (NVCs) was higher in both the KET and MXE groups. All of the measured urodynamic parameters and the associated statistical comparisons between groups are shown in Figure 3B,C. Both ketamine and methoxetamine induced bladder storage dysfunction, which was characterized by increased frequencies of frequency of voiding contractions (VCs) and NVCs, as well as decreased bladder capacity, inter-contraction interval (ICIs), and bladder compliance.

### 2.3. Drug Effects on Mucosal Structure and the Epithelium Barrier

**Figure 4:**
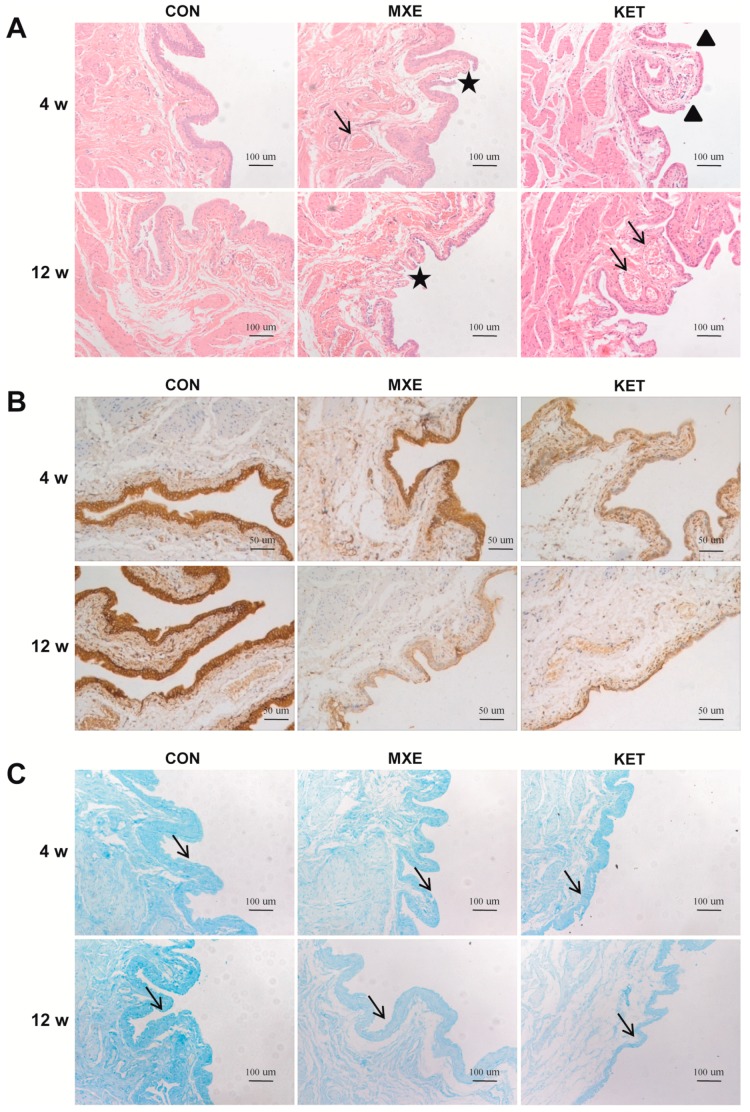
Mucosal injury in rat bladder tissue after MXE and KET administration. (**A**) Methoxetamine-treated rats showed mild tissue congestion (arrow) and interrupted continuity of mucosa (asterisks). Magnification: ×100, scale bar: 10 μm. The bladder tissues of ketamine-treated rats were characterized by mucosal laceration (triangles), papillary protrusions, and erythrocyte accumulation (arrows); (**B**) a decreased staining of *E*-cadherin was noted in the MXE and KET groups when compared with rats in the control group (magnification: ×200; scale bar: 50 μm); (**C**) alcian blue staining results. A reduced expression of acid mucopolysaccharide was observed in either MXE or KET groups (arrows, magnification: ×100; Scale bar: 100 μm). The representative images in three treatment groups (*n* = 6 in each group) were illustrated above.

Gross pathological changes in the bladder mucosa were evaluated by microscopy (Figure 4A). The bladders of methoxetamine-treated rats showed mild tissue congestion and mucosal degeneration, as well as a reduced number of urothelium layers. The bladder tissues of ketamine-treated rats displayed several abnormal characteristics, which included congestion, erythrocyte accumulation, neovascularization, a denuded urothelium or a mucosal laceration, papillary protrusions, and scattered mononuclear cells in perivascular regions.

Although not directly quantified, *E*-cadherin staining appeared decreased in methoxetamine- or ketamine-treated rats (Figure 4B), suggesting participation of epithelial barrier dysfunction in the pathogenesis of cystitis.

The alcian blue staining studies revealed lower GAG staining intensities in the MXE group and KET group as compared with the control group (Figure 4C).

### 2.4. Mast Cell Infiltration and Cytokine/Chemokine up-Regulation

**Figure 5:**
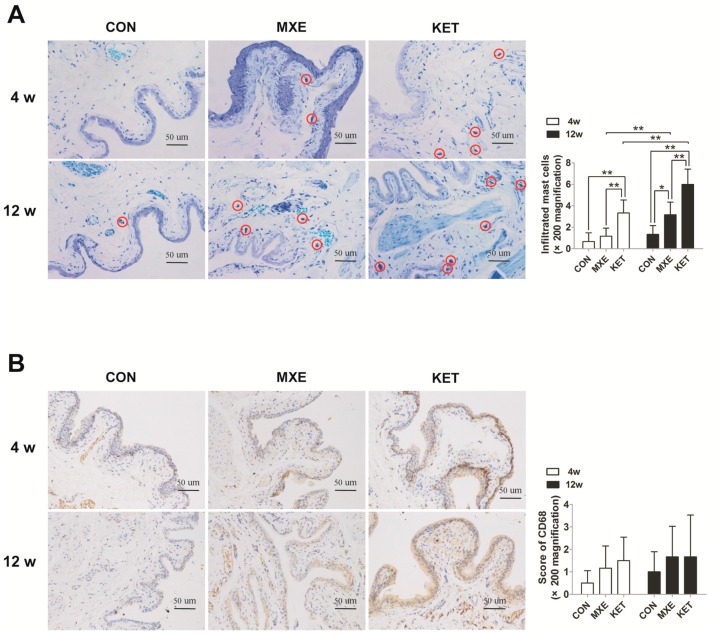
Mast cell and macrophage infiltration in rat bladder tissue after MXE and KET treatment. Representative graphs of toluidine blue-stained mast cells (**A**); and CD68 positive macrophages (**B**) in the three groups (*n* = 6 in each group). Magnification: ×200; Scale bar: 50 μm. Mast cell and macrophage infiltration was greater and tended to increase with drug exposure time in the MXE and KET groups (red circles indicate mast cells). Data were analyzed with two-way. * *p* < 0.05, ** *p* < 0.01.

The number of mast cells which stained with toluidine blue and turned purple upon degranulation and aggregation were significantly increased in drug treated group at 12 weeks and only significantly higher in KET treatment at fourweeks in the rat bladder tissues (Figure 5A). The infiltrating mast cells were concentrated in the mucosal, submucosal, and muscular layers of bladder tissue. Similarly, the mean percentage of CD68 positive cells in drug-treated rats seemed to be increased when compared with their percentage in control rats; however, the difference was not statistically significant (Figure 5B).

**Figure 6:**
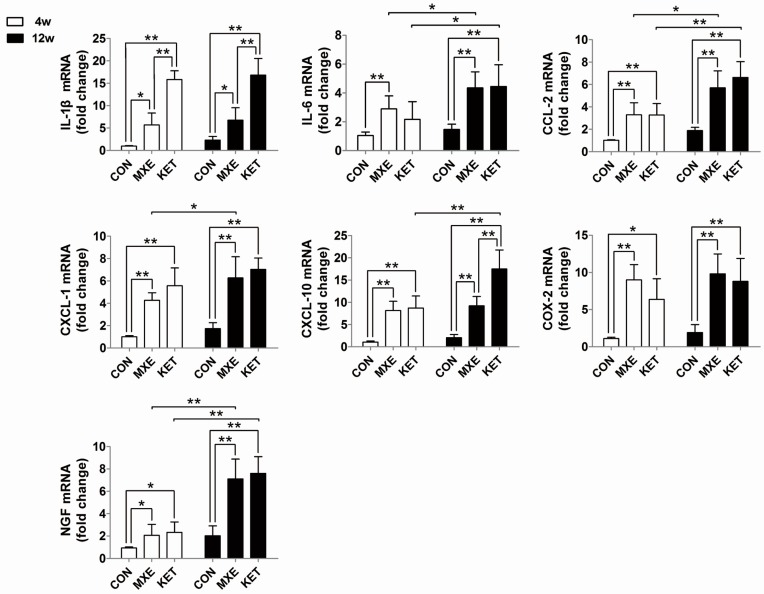
Relative levels of cytokine/chemokine gene expression of rats. The qRT-PCR analysis results for IL-1β, IL-6, CCL-2, CXCL-1, CXCL-10, COX-2, and NGF in bladder tissue of normal saline, methoxetamine and ketamine treated rats (*n* = 6 in each group). Data are expressed in mean ± SD, as the fold-change vs. the mean value in the control group at four weeks. Two-way ANOVA were used for analysis. * *p* < 0.05, ** *p* < 0.01.

Quantitative real-time PCR analysis was performed to detect changes in mRNA levels of cytokines and chemokines (IL-1β, IL-6, CCL-2, CXCL-1, CXCL-10, COX-2, and NGF). Our results demonstrated a dose- and time-dependent up-regulation of these cytokines/chemokines mRNA levels (Figure 6).

### 2.5. Matrix Deposition and Interstitial Fibrosis

**Figure 7:**
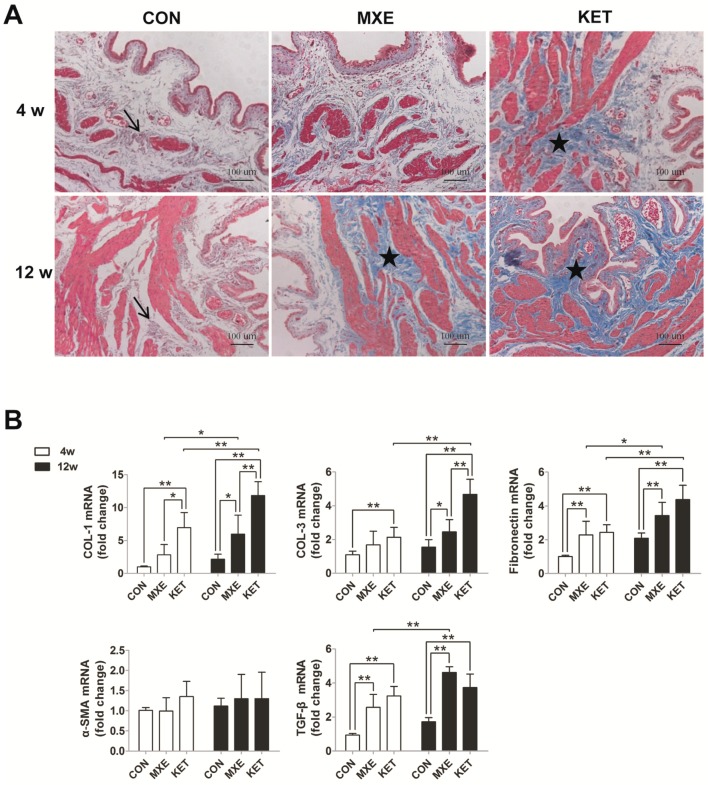
MXE and KET administration induced matrix deposition and interstitial fibrosis in rats after four and 12 weeks. (**A**) Representative graphs of Masson trichrome staining results in the different groups (*n* = 6 in each group). (magnification: ×100; scale bar: 100 μm). In the age-matched control group, only a few discrete light blue stained collagen deposits (arrows) were observed among the muscle bundles. Treatment with ketamine for four weeks or methoxetamine for 12 weeks caused significant increases in dense connective tissue and collagen deposits between the muscle bundles (asterisks); primarily in the submucosal and muscular layers. Bladder tissues from the 16-week KET group were characterized by the disappearance of lamina propria and submucosa, rather than by diffuse interstitial fibrosis; (**B**) expression levels of genes for collagen I (COL I), collagen III (COL III), fibronectin, α-SMA, and TGF-β. Data are expressed in mean ± SD, as the fold-change vs. the mean value in the control group at four weeks. Two-way ANOVA were used for analysis. * *p* < 0.05, ** *p* < 0.01.

**Figure 8:**
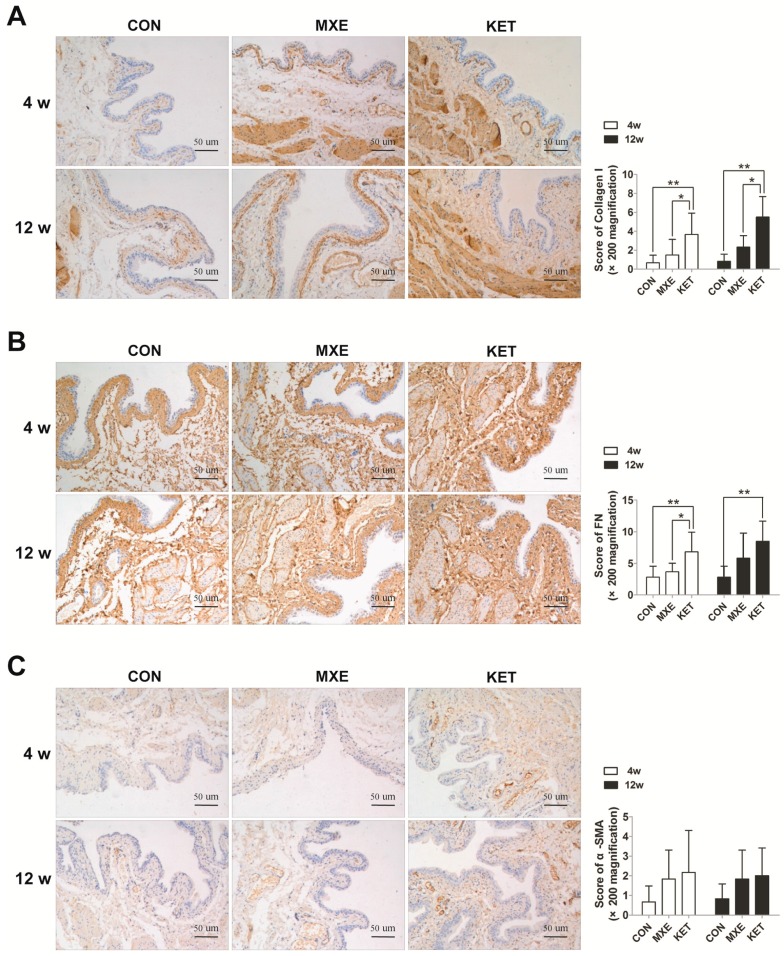
Immunohistochemistry of: collagen I (**A**); fibronectin (**B**); and α-SMA (**C**) in bladder tissue of normal saline, methoxetamine and ketamine treated rats (*n* = 6 in each group). A trend of increasing interstitial fibrosis with prolongation of exposure time was observed in the MXE group or KET group. Data are expressed in mean ± SD, as the fold-change vs. the mean value in the control group at 4 weeks. Two-way ANOVA were used for analysis. * *p* < 0.05, ** *p* < 0.01.

We further detected fibrotic damage in bladder tissue by Masson trichrome staining methods (Figure 7A), and also investigated the expression of fibrosis-related genes (Figure 7B) and proteins (Figure 8). Rats in the age-matched control group showed only a few discrete light blue stained collagen deposits in the muscle layers of their bladder tissue. In contrast, rats treated with ketamine for four weeks or methoxetamine for 12 weeks showed significant amounts of dense connective tissue proliferation and collagen deposition, primarily in their tissue submucosal and muscle layers. Of note, bladder tissues from the 12-week KET group were characterized by the disappearance of lamina propria and submucosa, instead of by diffuse interstitial fibrosis. Moreover, fiber strands which extended into the curly mucosa were a common finding, indicating that prolonged exposure to ketamine had resulted in a larger area of collagen infiltration. Consistent with these findings, the levels of mRNA expression for various fibrosis markers showed varying degrees of increase due to drug treatment. However, the expression level of alpha-smooth muscle actin did not significantly differ among the MXE, KET and control groups.

### 2.6. Direct Toxic and Pro-Inflammatory Effects on Bladder Epithelial Cells In Vitro

**Figure 9:**
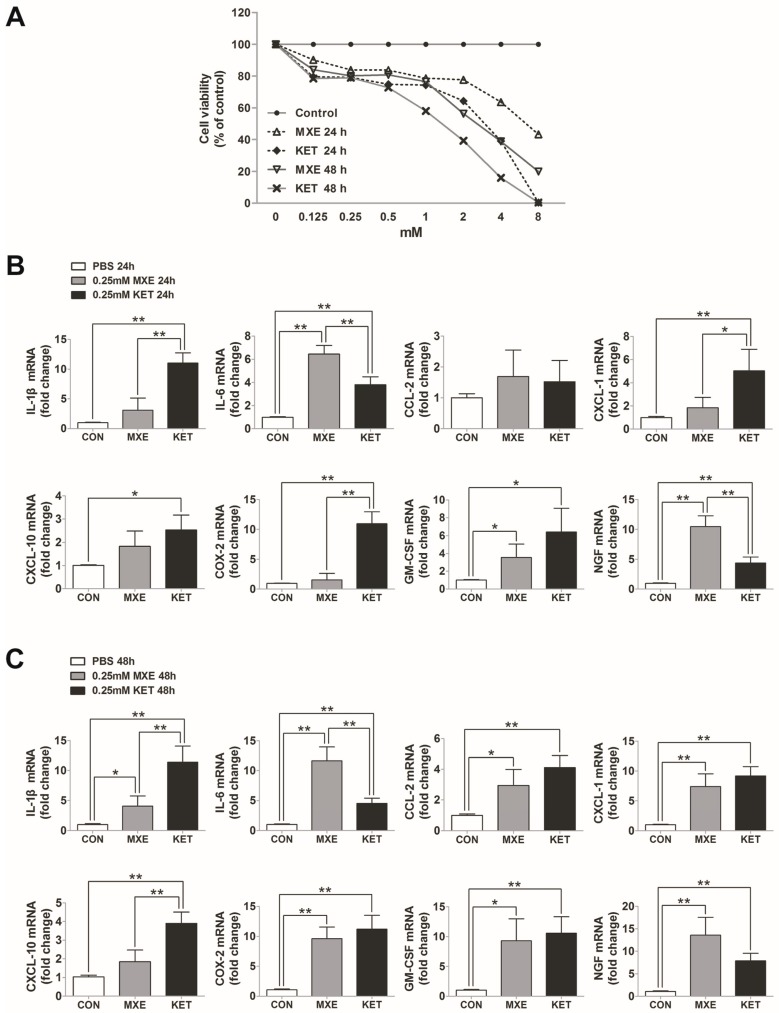
Direct toxic and pro-inflammatory effects on bladder epithelial cells in vitro. (**A**) CCK-8 assay results for SV-HUC-1 cell viability. Expression of genes for cytokines/chemokines, including IL-1β, IL-6, CCL-2, CXCL-1, CXCL-10, COX-2, GM-CSF, and NGF in SV-HUC-1 cells stimulated with methoxetamine (MXE) or ketamine (KET): for 24 h (**B**); or 48 h (**C**). Data are expressed in mean ± SD, as the fold-change vs. the mean value in the control group at four weeks. One-way ANOVA were used for analysis. * *p* < 0.05, ** *p* < 0.01.

To elucidate whether methoxetamine or ketamine had any direct toxic effect on bladder epithelial cells in vitro, SV-HCU-1 cells which had been stimulated with 0–8 mM ketamine or methoxetamine for 24 or 48 h were assessed for their viability with the CCK-8 assay (Figure 9A). The results showed that exposure to MXE or KET evoked a significant concentration-dependent and time-dependent reduction in viability.

We selected a 0.25 mM drug concentration for use in studying the effects of KET and MXE on pro-inflammatory molecule synthesis, because at that concentration, the SV-HCU-1 cells displayed a level of viability that was ~80% that of control cells. This allowed us to exclude the effects of interference resulting from the excessive release of cytokines/chemokines due to necrocytosis. Similar to results in the in vivo experiments, the expression levels of mRNA for cytokines/chemokines in SV-HUC-1 cells appeared to increase after the cells had been incubated with methoxetamine or ketamine for 24 or 48 h, respectively (Figure 9B,C). These findings confirmed that either MXE or KET could directly damage urothelial cells.

## 3. Discussion

Our results suggest that MXE and KET cause changes in the urination patterns of rats. Cystometry investigations showed that bladder capacity, and bladder compliance decreased, while the numbers of non-voiding contractions and urination frequency increased in the drug-treated rats. Histologic studies revealed damaged urothelium barriers, reduced amounts of glycosaminoglycans, but increased levels of congestion, inflammatory cell infiltration, and fibrosis in the bladders of drug-treated rats. Moreover, the expression levels of several cytokines in bladder tissue were significantly increased following MXE or KET exposure. In addition to its cytotoxic effects, MXE also stimulated human urothelium cells to generate cytokines. When taken together, these findings explain that MXE induces cystitis and impairs bladder function. In other words, MXE may not be “bladder friendly”.

This study is the first to investigate the impact of MXE on the rat bladder, and is also novel because urodynamic measurements have not been previously employed to measure the effects of MXE in animals. Various researchers have used cystometry to objectively evaluate the status of the lower urinary tract in animal models of ketamine-induced cystitis [11,16,17], and the results found in our KET-treated rats were consistent with the findings in those previous studies. The rats that experienced long term MXE exposure also displayed detrusor over activity and low bladder compliance. Thus, the urodynamic data suggest that MXE induces lower urinary tract symptoms similar to those caused by ketamine exposure.

Ketamine was found to cause damage in the bladder mucosal, submucosal, and muscular layers. Several previous studies have reported that ketamine administration resulted in a thinner or denuded epithelium, inflammatory cell infiltration, and vascular proliferation in the submucosal layer of bladder tissue, as well as collagen deposition in the muscular layer [16,18]. We found similar lesions in our MXE-treated rats. Moreover, results obtained in a previous study by Dargan et al. [14] support our histology findings, and also indicate that MXE is toxic to bladder tissue.

Glycosaminoglycans (GAGs) are distributed on the surface of the bladder mucosal epithelium. Our alcian blue staining studies revealed lower GAG staining intensities in the MXE group and KET group as compared with GAG staining intensity in the control group. As an important component of bladder epithelium barriers, GAGs protect the vesical mucosa from bacteria, molecules, and ions present in the urine. A decreased excretion of GAGs and defects in the epithelial permeability barrier are regarded as mechanisms of bladder pain syndrome/interstitial cystitis (BPS/IC). Moreover, a previous study showed that rats with ketamine-induced cystitis had reduced levels of glycoprotein GP51 and potassium in their urine [19].

*E*-cadherin is a glycoprotein found in cell-to-cell adherens junctions, and is crucial for maintaining epithelial morphology and polarity. *E*-cadherin expression is often repressed in patients with various bladder disorders associated with LUTS, such as BPS/IC, ketamine-induced cystitis (KC), and recurrent UTIs [20]. Jiang et al. [21] proposed that *E*-cadherin, involved in bladder dysfunction secondary to bladder outlet obstruction. In the present study, *E*-cadherin content in the urothelium became significantly reduced somewhat later after MXE or KET administration. Consistent with KC [12], the above findings suggest that MXE damages the bladder epithelium barrier by decreasing its GAGs content and thereby affecting cell adhesion.

Another important feature in the examined bladder specimens was evidence of mast cell infiltration. Activated mast cells play a dominant role in the mechanism of BPS/IC [22]. In previous studies, mast cell numbers were found to be related to bladder capacity in patients with KC, BPS/IC, and especially in patients with KC [9]. Our results concerning mast cell infiltration in the MXE-treated rats suggest the need for an additional study to investigate the relation between chronic inflammation and clinical symptoms. Strong evidence exists that mast cells undergoing activation and degranulation secrete significant amounts of pro-inflammatory mediators, and especially histamine and various cytokines.

We detected increased mRNA levels of numerous cytokines in bladder tissues obtained from the MXE group. Among those cytokines, IL-1β, IL-6, CCL-2, and NGF are widely regarded as biomarkers for BPS/IC [23]. Aside from cytokines, the mRNA levels of some representative chemokines such as CXCL-1, CXCL-10, and CCL-2 were also increased. Chemokines are known contributors to symptoms of bladder disorders, and Perters found remarkably reduced levels of chemokines CXCL-1, CXCL-10, and CCL-2 in patients who had received sacral neuromodulation therapy for BPS/IC [24]. COX-2 is another pro-inflammatory mediator and a key enzyme in the prostaglandin synthesis pathway. Additionally, COX-2 also plays an important role in overactive bladder, bladder inflammation, and BPS/IC. Juan et al. demonstrated that rats with bladder dysfunction had up-regulated COX-2 mRNA levels, and that treatment with a COX-2 inhibitor prevented those changes [11]. The evidence showing increased levels of pro-inflammatory mediators after long term MXE or KET administration suggests that the type of cystitis induced by MXE or KET is similar to interstitial cystitis.

Numerous animal studies have proven that long term ketamine treatment leads to bladder fibrosis [17], which is an important factor in bladder abnormalities such as decreased capacity, lower compliance, and impaired detrusor function. This is the first report of mRNA and protein levels being used to verify increased deposition of collagen and fibrotic products in the muscular layer of the MXE-treated rats bladder. Furthermore, the increased extracellular matrix production may be associated with irreversible bladder fibrosis. This change became more severe after a long term exposure to MXE, which could also aggravate LUTS and become a risk factor for upper urinary tract involvement.

No previous study has focused on how MXE affects bladder epithelial cells. In our study, we found that MXE and KET produced toxic effects in SV-HUC-1 cells in a time and dose-related manner. A study by Shen et al. showed that KET was cytotoxic to human urothelial cell lines [10]. Those findings are consistent with ours, and strengthened our results. We found that treatment with MXE increased the levels of pro-inflammatory mediators in human urothelial cells, and identified a similar trend regarding mediator expression in bladder tissue. These results suggest that the cytokines may not have been released simply as a result of chronic inflammation, but also due to the biological effect of MXE on urothelial cells.

Our study has several limitations: (1) Clinical investigation and data were not included in present study. The characteristics of MXE abusers awaits further studies; (2) The occurrence and development of bladder inflammation involve various cells. Our study solely estimated the expression of pro-inflammatory mediators in urothelial cells after MXE administration. However, we consider that it is still valuable for our purpose to understand the pro-inflammatory effect of MXE; (3) Only one dose, one preparation and one administration route were tested.

The potential harm resulting from MXE abuse has not been widely recognized. Lawn et al. conducted a survey of 427 MXE abusers who had once used ketamine, and found that 23% of them experienced LUTS [13]. This was close to the 26.6% incidence of ketamine-associated urinary symptoms found in another study. Furthermore, the frequency of MXE usage, but not ketamine usage, was shown to be associated with LUTS occurrence. Currently, only nine countries (Japan, the United States, and sevencountries in Europe) have unequivocally prohibited the use of MXE [25]. Purchasing MXE does not require any certificate or authorization, and with the help of the Internet, MXE is even easier to acquire than KET [5]. Botanas et al. published a report implying that both MXE and KET can be potentially abused by humans [26]. When considering the extensive popularity of ketamine in Asian and other countries, the potential for MXE or other KET-related substances to be abused as recreational drugs is a matter of serious concern.

## 4. Materials and Methods

### 4.1. Animals and Drug Administration

Thirty-six female Sprague-Dawley rats (180–210 g) were purchased from the Laboratory Animal Center of Southern Medical University (SMU) in Guangzhou China. The animals were randomly assigned to three groups which received a single daily intraperitoneal injection of 0.9% saline (control group), 30 mg/kg methoxetamine (MXE group) or 30 mg/kg ketamine (KET group) for a period of either 4 or 12 weeks. Ketamine hydrochloride solution (0.1 g/2 mL; Fujian Gutian Pharmaceutical Co., Ltd., Fujian, China) and methoxetamine solid (Hanxiang Biologicals Inc., Guangzhou, China) were approved for use in this study by the Food and Drug Administration of Guangdong Province, China. The rats were weighed each week to adjust the amount of drug they received. All experimental procedures were performed in accordance with the Guidelines for Laboratory Animal Care, and the study protocol was approved by the Institutional Animal Care and Use Committee of SMU (serial number: SCXK Guangdong 2014-0047, 12 November 2014).

### 4.2. Micturition Frequency Measurement

Micturition frequency was measured as previously described [20]. Briefly, each rat was placed alone in a metabolic cage for a period of acclimatization. Next, a piece of modified filter paper which had been immersed in CuSO_4_·5H_2_O for >40 min and then dehydrated at 160 °C for 30 min to promote conversion of CuSO_4_·5H_2_O to CuSO_4_ was stretched across the underside of the cage. Direct contact between falling urine and the paper immediately initiated hydration of the CuSO_4_, resulting in discoloration of the paper. Micturition frequency was determined by calculating the number of spots on the paper as determined using Photoshop software (version 13.0, Adobe Systems Incorporated, San Jose, CA, USA) in reversed color contrast phase. Overlapped urine spots with legible edges were considered to be a single urination incident. Micturition frequency was measured for a period of 3 h per day, for 3 consecutive days, and the mean value was determined via statistical analysis.

### 4.3. Urodynamic Investigations

The rats were anesthetized by injection with 20% urethane (1.0 g/kg); after which, they were immobilized in the supine position, and a 1.0 mm inner diameter epidural catheter was transurethrally inserted into the bladder. The other end of the catheter was connected to a pressure transducer and a micro-pump via a *T*-branch adaptor. After emptying the bladder with a syringe, the bladder was infused with 0.9% sterile saline at a steady rate of 12 mL/h (200 μL/min). When a consistent and reproducible pattern of micturition was established, digital intravesical pressure signals were continuously recorded for 60 min or at least 5 void cycles using a MMS Solar system (Medical Measurement Systems; Enschede, The Netherlands). The measured parameters included VCs, NVCs, bladder capacity, PVR volume (post-void residual volume), ICI, baseline pressure (lowest pressure between adjacent voids), threshold pressure (exact initial pressure that triggered a VC), micturition pressure (peak pressure during urination), and bladder compliance (the dilated bladder volume as determined from baseline/the increase in intravesical pressure).

### 4.4. Histological and Immunohistochemical Staining

After completing the urodynamic investigations, the rats were sacrificed and their bladder tissues were removed. Specimens of bladder tissue for histologic staining were fixed in 4% paraformaldehyde; after which, they were embedded in paraffin and cut into 4 μm sections for analysis. Sections for histopathologic evaluation were stained with hematoxylin and eosin (H&E, Servicebio, Inc., Wuhan, China), alcian blue, masson’s trichrome and toluidine blue. Epithelial integrity was determined by *E*-cadherin expression. Staining for CD68 was performed to analyze macrophages aggregates present in the urinary bladders. Briefly, the tissue sections were deparaffinized and immersed in 3% H_2_O_2_ for 30 min to quench endogenous peroxidase activity. After being blocked with 2% BSA, the sections were incubated with *E*-cadherin (1:100, Santa Cruz Biotechnology; Santa Cruz, CA, USA), CD68 antibody (1:200, Santa Cruz Biotechnology), collagen I antibody (1:100, Boster; Pleasanton, CA, USA ), fibronectin antibody (1:500, Abcam, Cambridge, UK) or α-SMA antibody (1:200, Abcam) overnight at 4 °C. Following incubation with the primary antibodies, the tissue sections were incubated with the appropriate biotinylated secondary antibody (1:5000, Cwbio, Beijing, China) for 30 min; after which, they were incubated with 3,3′-diaminobenzidine and lightly counterstained with hematoxylin. Quantitative digital image analysis was performed using Image-Pro Plus 6.0 software (Media Cybernetics, Inc., Rockville, MD, USA), and representative images were selected for presentation in this report.

### 4.5. Cell Culture and Treatment

SV-HUC-1 cells (an immortalized human uroepithelial cell line infected with the SV40 virus) were cultured in DMEM/F12 medium containing 10% at 37 °C in a 5% CO_2_ atmosphere. Prior to treatment, the cells were transferred to serum-free medium and incubated overnight to synchronize their growth; after which, they were treated with ketamine or methoxetamine solution at the indicated concentration for the designated time period.

### 4.6. CCK-8 Assay

SV-HUC-1 cells (100 µL containing 5000 cells) were seeded into individual wells of a 96-well plate, and then pre-incubated for 24 h to allow their adherence. The cells were then incubated with 0.25 mM ketamine or methoxetamine for 24 or 48 h, respectively. Following incubation, 10 μL of CCK-8 solution (Dojindo Laboratories, Kumamoto, Japan), which serves as a tetrazolium salt colorimetric assay, was added to each well of the plate and incubated with the cells for 2 h. Next, a microplate reader (Tiangen Biotech Co., Ltd., Beijing, China) was used to measure the absorbance of each well at 450 nm.

### 4.7. Real Time-PCR

Total RNA was extracted from rat bladder tissue and SV-HUC-1 cells using RNAiso Plus reagent (Takara Bio Inc., Otsu, Shiga, Japan). Complementary DNA (cDNA) was synthesized using the PrimeScript™ RT reagent Kit (Takara Bio Inc.), and genomic DNA was eliminated according to the manufacturer’s instructions. Next, qRT-PCR was performed using an Applied Biosystems 7500 Real Time PCR System and SYBR^®^ Premix Ex TaqTM II (Takara Bio Inc.). The sequences of the primer pairs were designed by Invitrogen (Paisley, UK) (Table 1), and primer-blast programs were used to ensure the specificity of the amplified fragments. The cycle threshold (*C*_t_) of various genes was determined, and changes in gene expression were calculated using the delta-delta *C*_t_ method. The results are expressed as a ratio relative to GAPDH. Allof the PCR experiments were repeated individually at least 3 times.

### 4.8. Statistical Analysis

All data were analyzed using SPSS for Windows, Version 13.0. Chicago, IL, USA: SPSS Inc., and results are expressed as the mean ± SD. Multiple group comparisons were performed using analysis of variance (ANOVA), and the LSD test was used for pair-wise comparisons (equal variances assumed). Otherwise, the Dunnett’s *t*_3_ test was preferred. For all analyses, two-sided *p*-values < 0.05 were considered statistically significant.

## 5. Conclusions

Methoxetamine is a novel synthetic psychoactive ketamine analog. Considering the increasing cases of ketamine-induced cystitis, it is crucial to explore the effect of MXE to urinary system. Our results of cystometry and histologic examinations indicate that long term MXE treatment can induce bladder dysfunction and inflammation in rats. In vitro, methoxetamine was confirmed to produce direct toxic and pro-inflammatory effects on human urothelial cells. MXE-associated bladder impairment may be similar to ketamine-induced cystitis. Our findings not only reveal the influence of MXE on the bladder, but also suggest that MXE-associated bladder impairment may be similar to ketamine-induced cystitis.

## Figures and Tables

**Table 1:** Real-time reverse transcription-polymerase chain reaction (RT-PCR) primers.

Gene (Source: Rat)	Forward Sequence (5′–3′)	Reverse Sequence (5′–3′)
GAPDH	CAGGGCTGCCTTCTCTTGTG	GGTGATGGGTTTCCCGTTGA
IL-1β	TGATGAAAGACGGCACACCC	ATGTCCCGACCATTGCTGTT
IL-6	CTCTCCGCAAGAGACTTCCA	ATACTGGTCTGTTGTGGGTGG
CCL-2	CACTCACCTGCTGCTACTCA	CCTTATTGGGGTCAGCACAGA
CXCL-1	CTCCAGCCACACTCCAACAG	GACTTCGGTTTGGGTGCAGT
CXCL-10	TGAAAGCGGTGAGCCAAAGA	CTAGCCGCACACTGGGTAAA
COX-2	GTTGCTGGGGGAAGGAATGT	AGCATCTGGACGAGGCTTTT
NGF	CGCTCTCCTTCACAGAGTTTT	CTGCCTGTACGCCGATCAAA
Collagen I	AAGGGAGGAGAGAGTGCCAA	GTCTCTTGCTTCCTCCCACC
Collagen III	TGCAATGTGGGACCTGGTTT	GGGCAGTCTAGTGGCTCATC
Fibronectin	GGGGAAGAAAAGGAGCCCAG	GGACCCCTGAGCATCTTGAG
α-SMA	CATCCGACCTTGCTAACGGA	AATAGCCACGCTCAGTCAGG
TGF-β	GACTCTCCACCTGCAAGACC	GGACTGGCGAGCCTTAGTTT
GAPDH	AACGGATTTGGTCGTATTGGG	CCTGGAAGATGGTGATGGGAT
IL-1β	ATGGCTTATTACAGTGGCA	GTAGTGGTGGTCGGAGATT
IL-6	ATGAGGAGACTTGCCTGGTGAA	CAATCTGAGGTGCCCATGCTAC
CCL-2	GCTCATAGCAGCCACCTT	GGAATCCTGAACCCACTT
CXCL-1	CAAACCGAAGTCATAGCCACA	TTCTCCTAAGCGATGCTCAAA
CXCL-10	AAAAGAAGGGTGAGAAGA	AGTGCCAGGGTAGAGTTA
COX-2	AGTCCCTGAGCATCTACGGT	AAAGGTGTCAGGCAGAAGGG
NGF	GTTTAGCACCCAGCCTCC	GCTCTTCTCACAGCCTTCC

## Abbreviations

MXE	methoxetamine
KET	ketamine
LUTS	lower urinary tract symptoms
VC	frequency of voiding contractions
NVC	frequency of non-voiding contractions
GAGs	glycosaminoglycans
BPS/IC	bladder pain syndrome/interstitial cystitis
KC	ketamine-induced cystitis
